# Progress in the Development of Graphene-Based Biomaterials for Tissue Engineering and Regeneration

**DOI:** 10.3390/ma15062164

**Published:** 2022-03-15

**Authors:** Chao Chen, Yuewei Xi, Yunxuan Weng

**Affiliations:** 1 College of Chemistry and Materials Engineering, Beijing Technology and Business University, Beijing 100048, China; 2030401001@st.btbu.edu.cn; 2 Beijing Key Laboratory of Quality Evaluation Technology for Hygiene and Safety of Plastics, Beijing Technology and Business University, Beijing 100048, China

**Keywords:** graphene-based biomaterials, tissue engineering, soft tissue, biocompatibility

## Abstract

Over the last few decades, tissue engineering has become an important technology for repairing and rebuilding damaged tissues and organs. The scaffold plays an important role and has become a hot pot in the field of tissue engineering. It has sufficient mechanical and biochemical properties and simulates the structure and function of natural tissue to promote the growth of cells inward. Therefore, graphene-based nanomaterials (GBNs), such as graphene and graphene oxide (GO), have attracted wide attention in the field of biomedical tissue engineering because of their unique structure, large specific surface area, good photo-thermal effect, pH response and broad-spectrum antibacterial properties. In this review, the structure and properties of typical GBNs are summarized, the progress made in the development of GBNs in soft tissue engineering (including skin, muscle, nerve and blood vessel) are highlighted, the challenges and prospects of the application of GBNs in soft tissue engineering have prospected.

## 1. Introduction

**Figure 1:**
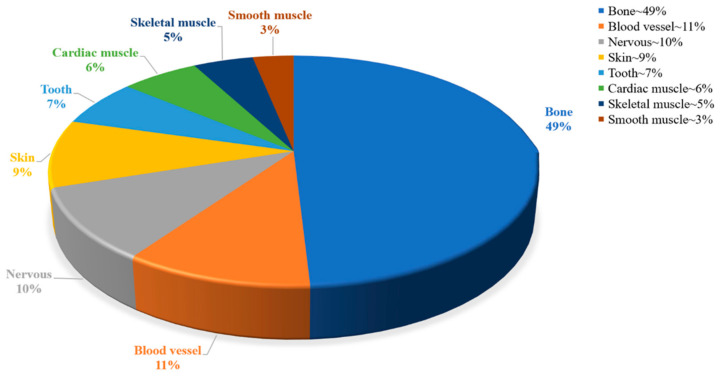
Percentage of correlation based on different types of tissue engineering.

The broad definition of soft tissue includes a variety of tissues such as skin, muscle, nerves and blood vessels, which usually have the function of enclosing, supporting or connecting body structures and organs [1]. However, due to the occurrence of factors such as disease, trauma, and aging, non-self-healing behavior may occur after soft tissue injury. Current organ transplantation is the main technique for treating this damage, and the limitations of organ transplantation have been a major concern due to the shortage of transplantable organs and the rejection of these organs. Tissue engineering has emerged as an important new medical strategy to overcome the current clinical therapeutic problems of repairing and regenerating damaged sites. Tissue engineering, which aims to produce regenerative tissue for clinical treatment, has attracted great attention in the past 30 years [2]. In 1988, tissue engineering was defined as the use of principles and methods of engineering, materials science, and life sciences to simulate the structure and function of the target tissue to develop a biologically active tissue replacement to reconstruct, maintain, and improve the physiological function of the affected tissue. Tissue engineering can restore, maintain, or improve the function of a tissue or entire organ by using a combination of cells, biomaterials, and appropriate biochemical factors [3,4]. In recent years, tissue-engineering-based therapies have been widely applied to bone [5], skin [6], cardiovascular [7], nerve [8], dental [9] and muscle [10] tissues (Figure 1). The wound-healing and orthopedic applications have been approved for clinical trials by the Food and Drug Administration and are already available for commercial use [11].

**Figure 2:**
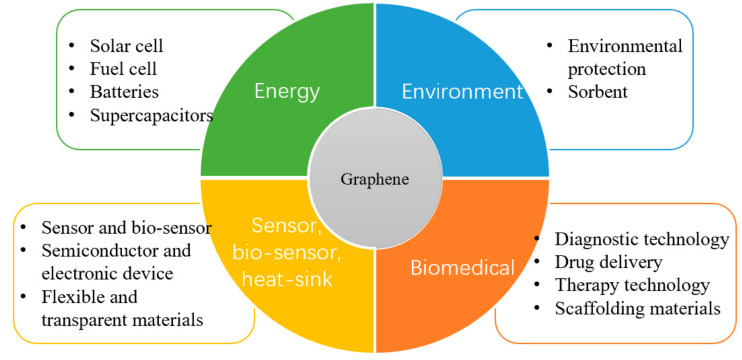
Graphene application in research.

**Figure 3:**
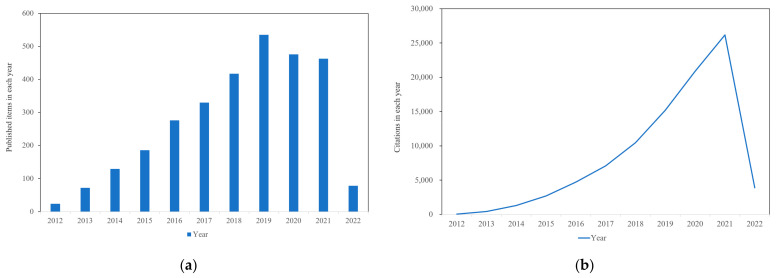
Trends in (**a**) publications and (**b**) citation frequency in graphene-based tissue engineering (data from Web of Science; Search strings: “graphene” and “tissue engineering”).

The excellent properties of graphene make it widely used in energy, environment, biomedicine, sensors and more (Figure 2). Especially in the biomedical field, graphene-based nanomaterials (GBNs) have become candidates for tissue engineering applications such as wound healing, stem cell engineering, regenerative medicine, cell growth and differentiation due to their excellent mechanical strength, electrical conductivity, and various two-dimensional (2D) and three-dimensional (3D) structures [12,13]. It can be used as reinforcement material to construct tissue engineering scaffolds such as hydrogel, film, fiber and foam. At the same time, the excellent physical properties of graphene can provide functional bioactive surfaces, which have great potential in the field of tissue engineering when it exists in the form of composite materials [14]. So far, GBNs have been extensively explored in bone, nerve, heart, cartilage, musculoskeletal and skin/adipose tissue engineering [15]. To describe the importance of graphene, we used Web of Science to investigate the development trend of publication and citation rate of graphene in the field of tissue engineering, and it can be seen that it is in a state of continuous increase (Figure 3a,b). Although there is growing evidence that graphene materials have broad application prospects in the biomedical field, their potential harm to humans and the environment has attracted attention [16]. At present, the toxicity evaluation results are still controversial, indicating that despite many exciting results, the clinical application of these new materials is still a long way off.

**Figure 4:**
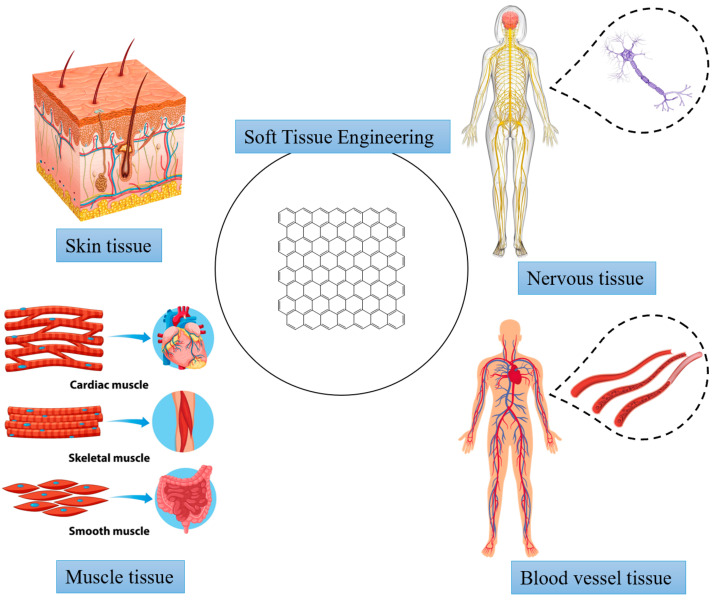
Applications in soft tissue engineering.

In this review, we study the application of GBNs in soft tissues (including skin, muscle, nervous and blood vessels) from the perspective of tissue engineering (Figure 4). Firstly, we discuss the structure and properties of GBNs and analyze their biocompatibility in soft tissue. Then we briefly review the latest research progress of GBNs application in soft tissue engineering. Finally, graphene nanocomposites as the next generation of soft tissue engineering materials are summarized and prospected.

## 2. Structure and Properties of GBNs

**Figure 5:**
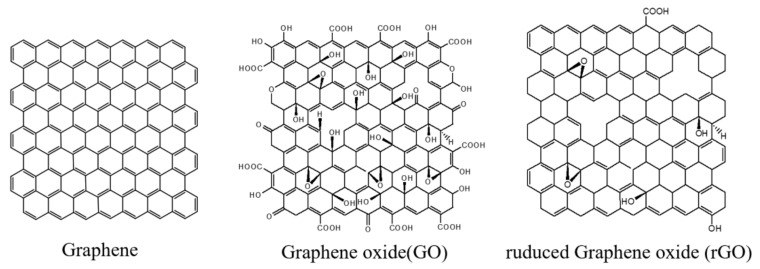
Schematic diagram of GBNs.

GBNs are excellent scaffolds for tissue engineering and have been successfully applied in tissue engineering research [17]. These carbon nanomaterials, due to their similar size, are considered analogs of extracellular matrices such as collagen fibers [18]. Graphene-based nanomaterials come in a variety of functionalized forms, mainly including graphene oxide (GO) and reduced graphene oxide (rGO) (Figure 5). These novel nanomaterials have unique properties such as unique surface properties, improved biocompatibility, photothermal effects, pH responsiveness, and a broad spectrum of antibacterial activity [19].

**Figure 6:**
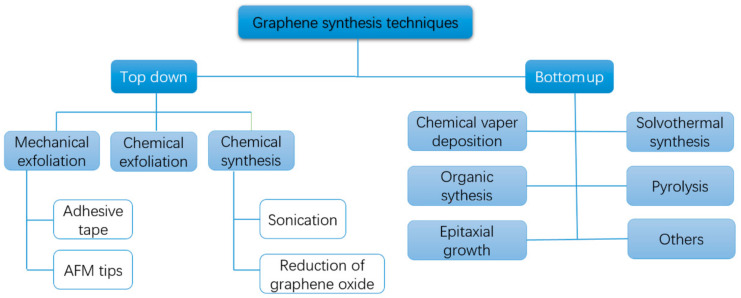
The synthesis method of graphene.

Graphene belongs to the basic structure of GBNs [20]. So far, graphene synthesis methods have been divided into two main categories: “bottom-up” and “top-down” methods (Figure 6). The first category is to destroy the van der Waals force between the graphite layers under the action of external forces, and the graphite sheet layer is stripped down; the other category is the synthesis of various GBNs by chemical reactions such as epitaxial growth, chemical vapor deposition and organic synthesis [21]. The carbon atoms in graphene exist in the form of sp^2^ hybridization, forming a huge π-π conjugated system in which electrons can move freely [15]. Therefore, graphene has a two-dimensional planar structure with high specific surface area and strong mechanical properties (130 GPa breaking strength, 32 GPa elastic modulus and 1 TPa young’s modulus) [22] and exhibits excellent thermal conductivity ((5300 W m^−1^K^−1^), electrical conductivity (10^4^ S/cm) and ultra-high electron mobility (250,000 cm^2^/Vs) at room temperature [23]. The thickness of monolayer graphene is about 0.34 nm [24]. Due to the strong C-C bond in the same plane, its hardness is higher than diamond, but contrary to diamond, it becomes a soft material through the interlayer bond of weak van der Waals force [25]. At the same time, graphene has a large specific surface area (2630 m^2^/g), enabling it to adsorb or bind more materials to achieve functionalization [25,26].

GO is a derivative of graphene. This highly oxidized monolayer of graphene contains a large number of oxygen-containing active groups, such as carboxyl group (-COOH), hydroxyl group (-OH), carbonyl group (-C=O) and epoxy group (-O-) [27]. The epoxy group and hydroxyl group are mainly located on the base plane of GO. Carbonyl and carboxyl groups are usually distributed at the edge of go, which has good water solubility and stability, making it more suitable for cell-surface attachment, proliferation and differentiation [28,29]. More importantly, these oxygen-containing functional groups of GO allow researchers to further their chemical functionalization, making GO an ideal platform for biomolecular regulation, which is a key factor in its biologically related applications [30]. At the same time, GO is amphiphilic and can be used as a surfactant. In addition, it has been proved that GO composite hydrogels can gel in an acidic medium and undergo a gel-sol transition in alkaline conditions [31]. Tumor tissue is generally more acidic (pH ~6.8) than normal cells (pH 7.4) [32]. At low pH conditions, the hydrogen bond between the drug and GO weakens each other, releasing the internal drug part. This property of GO has been incorporated into the design of pH-sensitive drug delivery systems.

Although oxygen-containing functional groups increase the stability and chemical reactivity of graphene in solution, the presence of functional groups produces high defect density, which may reduce conductivity, mechanical and optical properties [33]. The reduction of GO functional groups forms rGO. Compared with GO, rGO restores the conductivity and photothermal effect of graphene to a certain extent, and rGO is more hydrophobic than GO [34].

**Figure 7:**
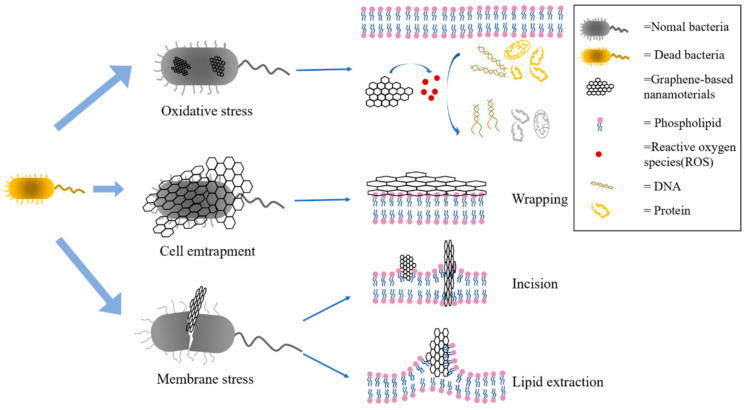
Antibacterial mechanism.

One of the advantages of GBNs in tissue engineering is their broad spectrum of antibacterial activity. Pathogenic microbial infection is one of the biggest public health problems in the world. With the abuse of fungicides and antibiotics, microorganisms gradually develop strong resistance to typical antibiotics, which is not conducive to follow-up treatment [35]. Therefore, it is of great significance to develop antimicrobial agents with different antibacterial mechanisms. GBNs, due to their special physical and chemical properties and unique antibacterial mechanism, has a good antibacterial effect on human pathogens such as *Escherichia coli* and *Staphylococcus aureus* (related to infections in different parts of the human body), *Streptococcus aeruginosa*, *Porphyromonas gingivalis* and *Candida albicans* (related to oral diseases), *Pseudomonas aeruginosa* and *Klebsiella pneumoniae* (a typical pathogen of hospital infection), *Salmonella typhi* (related to gastrointestinal diseases) and other human pathogens, and have great potential to become new antibacterial agents [36]. Some studies on the antibacterial activity of carbon nanotubes, graphene, GO, and rGO against *Escherichia coli* and *Staphylococcus aureus* have shown that rGO has the strongest antibacterial activity [25] (Figure 7). There are three main antibacterial mechanisms of GBNs: One is membrane stress, that is, GBNs such as GO and rGO have sharp edges, which cause physical damage to the cell membrane after direct contact with the cell membrane, causing leakage of intracellular substances, and ultimately leading to cell death [37]; the second is oxidative stress, that is, the ROS (Reactive oxygen spices) produced by GBNs causes an imbalance between oxidation and antioxidant, which interferes with the metabolism of bacteria, destroys the cellular structure, and ultimately leads to the loss of bacterial vitality [38]; the third is cell entrapment, that is, through physical enveloping, the bacterial cells are isolated from the surrounding environment, so that they cannot exchange substances with the environment, hinder their metabolic processes, and then inhibit the proliferation of bacterial cells [39]. While GBNs show antimicrobial properties, they tend to congregate due to strong intra-surface interactions, which limits their surface area and function. Therefore, to reduce agglomeration and improve antibacterial activity, graphene has been functionalized and surface modified to form nanocomposites by combining with other nanomaterials or biologically active substances (such as metal or metal oxide nanomaterials) to achieve an enhancement effect [40,41].

The photothermal effect of GBNs can be combined with antibacterial activity for synergistic antibacterial treatment. Under near-infrared (NIR) irradiation, GBNs absorb NIR light and converts it into heat, thereby killing bacteria through local heating [42]. At the same time, due to the influence of light absorption, temperature rise and atomic vibration, the non-covalent bond interaction on the GBNs surface is weakened [43]. Therefore, GBNs can not only be directly applied to photothermal therapy but also achieve controlled release of drugs, thus promoting cell differentiation and growth.

## 3. Biocompatibility of GBNs

Biocompatibility refers to the ability of a material to interact with cells, tissues or the body without causing damage [15]. Extensive studies have been conducted to elucidate the clinical biocompatibility of GBNs in vivo and in vitro, which is essential to confirm suitability for clinical use. However, the effects of GBNs on biological cells are that in some cases, they are biologically compatible and in others, they are toxic to cells. Although graphite is a well-studied naturally occurring carbon allotrope, Gr, GO, and rGO are artificially prepared materials, and information on GBNs toxicity in vitro and in vivo is far from complete as the size and oxidation state of GBNs vary [44,45]. The interaction of living cells with GBNs depends mainly on their transverse size, the number of layers, purity, hydrophilicity, surface chemistry, and dosage [46,47]. These properties vary greatly depending on the raw material, the method of synthesis, and the degree of functionality. Since it is difficult to draw accurate conclusions, we intend to guide future studies by comparing relevant studies.

The surface chemistry of graphene plays an important role in determining its cytotoxicity, which can be significantly reduced by functionalizing GBNs with biocompatible polymers or molecules. It was found that primitive graphene was more cytotoxic than carboxylated fossil graphene (G-COOH). Hydrophobic primitive graphene aggregated on the cell membrane at high concentrations, destroying cytoskeleton arrangement and deforming cells, while hydrophilic G-COOH at low concentrations was highly internalized and did not seem to affect cell functions [16]. Curley et al. [48] found that sulfonated GO was non-toxic within the concentration range of 0.1–10 mg/mL. Modifications such as carboxylation and dextran conjugate have also been shown to reduce the cytotoxicity of graphene by increasing the hydrophilicity of graphene derivatives [49]. In addition, polyethylene glycol (PEG) modification is probably the most widely used technology to improve biocompatibility and solubility. GO and rGO were functionalized with PEG and bovine serum albumin (BSA), and both molecules greatly reduced the cellular viability loss and genotoxic effects of GBNs, with GO-PEG showing the greatest improvement [50].

GBNs have a dose-dependent toxic effect on cells, which varies with different cell types. Siew et al. [51] found that the toxicity of GO/TiO_2_ nanocomposites on human lung fibroblasts and HaCaT cells depended on the exposure time and dose; that is, higher Gr concentration and longer exposure time corresponded with poor cell survival rate. Qiu et al. [52] found that the threshold of toxicity of graphene quantum dots to bone marrow mesenchymal stem cells was 50 μg/mL. Mazaheri et al. [53] found that human bone marrow mesenchymal stem cells (HMSCs) cultured on chitosan and GO (1.5 wt%) -chitosan composite layer had similar proliferation rates, and the cytotoxicity was significantly increased when GO content was 3 wt% and 6 wt%. Therefore, according to the literature, it can be concluded that the concentration of graphene and GO at 50 μg/mL, rGO at 60 μg/mL, and 1.5 wt% are the safest for most cells [54].

Another factor contributing to cytotoxicity is lateral size. Small particles of GO are highly toxic because of their small size, sharp edges, and ease to penetrate the cell membrane into the cytoplasm, resulting in cell membrane damage and leakage of cytoplasmic contents; GO tablets larger than 200 nm are less toxic because they do not penetrate cell membranes [55]. However, small GO particles are easily removed [56]. Akhavan et al. [57] reported that rGO nanosheets with smaller transverse sizes (11 ± 4 nm) had higher cytotoxicity than rGO nanosheets with larger transverse sizes (3.8 ± 0.4 μm). Therefore, the size of GO should be at the optimal size, which cannot penetrate the cell membrane and can be easily cleared from internal organs [58].

Therefore, the toxicity of GBNs can be reduced to a greater extent through functionalization, appropriate oxidation, reasonable design of shape and size or reduction of dosage.

In addition to in vitro studies, in vivo experiments were conducted to study the toxicity of graphene. Generally, after intravenous injection, GBNs mainly accumulate in lung, liver and spleen tissues in a dose-dependent manner, causing pathological damage to lung and liver tissues. In the lungs, high doses of GBNs lead to fibrosis, inflammation, and severe pulmonary vascular occlusion, accompanied by platelet thrombosis. In vivo circulation time, blood compatibility, persistence, biodegradation and the fate of by-products are all areas that require detailed study. In particular, the interaction between GBNs and the immune system remains unclear. Surface functionalization and coating with copolymers and surfactants can potentially improve the biocompatibility and biodistribution of different GBNs while maintaining their superior ability as drug delivery systems [50]. Therefore, more comparative studies are needed to evaluate the clearance capacity and biodegradability of GBNs. However, most studies only assess the safety of GBNs in a short period, and little is known about the long-term safety of these nanomaterials. This will be crucial for biomaterials used in tissue engineering.

## 4. Applications of GBNs in Soft Tissue Engineering

The main components of soft tissue are elastin and collagen fibers. Elastin has the greatest linear elasticity, while collagen fibers are the main loading elements that directly affect the physical and mechanical properties of soft tissues, which together determine the biomechanical properties of soft tissues [1]. The primary purpose of tissue engineering is to develop appropriate biomaterials to simulate the biological environment, provide appropriate mechanical properties for cells, facilitate cell migration, differentiation, proliferation and deposition, and at the same time carry out functional changes according to different environmental requirements [59]. Given its ease of functionalization and high mechanical strength, stiffness and conductivity, GBNs have attracted great interest in the field of tissue engineering. From the perspective of tissue engineering, the following four parts of soft tissue affected by GBNs are introduced: skin, muscle, nerve and blood vessel (Table 1).

### 4.1. Skin

As the largest organ of the human body, the skin mainly acts as a barrier and controls the entry and exit passage of various substances, thereby protecting the inside of the body from the influence of the external environment [64]. However, due to the elastic and soft nature of the skin, it is on the outermost part of the human body and can easily be damaged by disease or trauma [65]. Despite the skin’s ability to repair itself, wound care is still necessary to prevent infection and dryness, reduce pain, protect the injured area, speed up the healing process, and avoid scarring, especially for large, open wounds [66]. It is important to note that infection of the wound can delay healing and even lead to death. The global market for advanced wound care products is growing rapidly, from approximately $12 billion in 2020 to $18.7 billion by 2027 [67].

Currently, the pathology of wound healing is well studied. The skin wound healing process is divided into four overlapping stages: hemostasis, inflammation, proliferation, and remodeling of the tissue matrix. Different wound healing stages have their specific processes at the molecular and physiological levels, and any maladjustment at any stage will increase the risk of chronic refractory skin trauma, with increasing morbidity and mortality. Although skin grafts are the most commonly used method of repairing irreversible skin damage, they have defects that are prone to proliferation, infection, and scarring, so skin tissue engineering has developed well in the field of wound healing [68]. Wound dressings are necessary for wound healing, while traditional wound dressings such as gauze, bandage and cotton cloth are difficult to fit the wound surface. They have the disadvantages of poor absorption capacity and no antibacterial effect and are prone to tissue adhesion causing secondary damage when replaced. Therefore, a modern new wound dressing has been developed, which has the advantages of better biocompatibility and controllable mechanical properties, and the appropriate surface microstructure and biochemical properties can promote cell adhesion, proliferation and differentiation, in addition to supporting small molecule drugs to achieve multifunction [69,70]. Commonly used modern new wound dressings mainly include films and hydrogels. As a result, GBNs in the form of thin films or hydrogels have become candidate materials for wound dressing applications.

**Figure 8:**
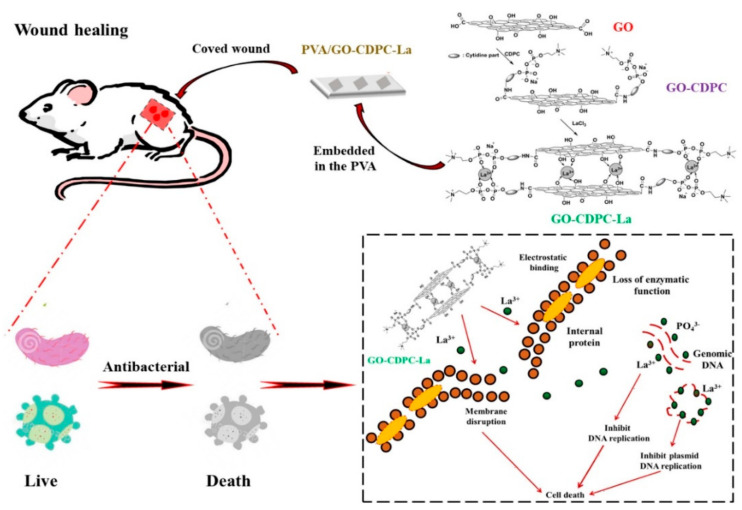
Schematic illustration of PVA antibacterial film embedded with GO-CDPC-La for antibacterial wound dressings. Data reproduced with permission from Ref. [74].

Film dressings are mainly composed of biomedical films and viscous materials, generally made of polyvinyl alcohol, polyethylene, polyurethane, polyacrylonitrile, polytetrafluoroethylene, polylactic acid and siloxane elastomers. GBNs themselves can promote wound healing while providing bactericidal ability, destroying the DNA structure of the microbial membrane and preventing proliferation, thereby improving wound healing rates. Mahmoudi et al. [71] prepared chitosan/polyvinylpyrrolidone (PVP) fiber film containing 2% GO nanosheets by electrostatic spinning method and used it as temporary skin graft material. Compared with the control group (sterile gauze), fibrous membranes containing 1.5% and 2% GO showed the greatest improvement in wound healing. At 14–21 days after surgery, no scar was found in the skin of the treated group, and the wound healing rate was 92%. Low concentrations of GO and rGO stimulate the synthesis of intracellular reactive oxygen species and reactive nitrogen, thereby inducing angiogenesis. Thangavel et al. [72] developed an isabgol (Isabella fruit) nanocomposite stent (Isab + rGO) loaded with rGO. Compared with Isab stent, Isab + rGO stent has a large liquid absorption capacity and promotes angiogenesis, collagen synthesis and deposition of therapeutic wounds. Since GO contains a large number of -COOH, -OH groups, and a unique sheet structure, mechanical and biological properties can be promoted by providing binding sites. Li et al. [73] used the antioxidant N-acetylcysteine (NAC) to load GO for sustained release and combined with type I collagen (Col) to prepare the composite film, which can continuously release the antioxidant NAC and have better mechanical properties and stronger water retention, to obtain the hybrid film for skin regeneration without a scar. Liu et al. [74]prepared polyvinyl alcohol/graphene oxide-citicoline sodium-lanthanum (PVA/GO-CDPC-La) film by solution intercalation method (Figure 8), which modified GO by antibacterial CDPC to improve the dispersion of water-based polymers while providing more adsorption sites for La^3+^ ions and enhancing the antibacterial properties of the membrane, which can significantly reduce the risk of wound infection. Jian et al. [75] made their antibacterial agent polyhexamethylene guanidine hydrochloride (PHMG) grafted GO (MGO) and introduced it into the bilayer thermoplastic polyurethane TPU composite porous membrane. The MGO-TPU bilayer membrane can still maintain antibacterial performance for a long time after 30 days of shaking and washing in PBS buffer for 30 days, which is significantly better than PHMG-TPU and GO-TPU systems alone, and the MGO-TPU bilayer membrane promotes the formation of re-epithelialization during wound healing. It speeds up the healing of the wound. Esmaeili et al. [64] encapsulated polyurethane/cellulose acetate (PU/CA) electrospun nanofiber film with curcumin and rGO/Ag at the same time, and the oxygen-containing functional groups on the base surface and edge of the rGO oxide film can be used as chemical anchor sites for nucleation of antibacterial AgNPs (Ag nanoparticles) to prevent the agglomeration of AgNPs, which not only improved the tensile strength of the scaffold but also improved its synergistic antibacterial properties. Li et al. [76] prepared rGO-coated gold nanoholes modified polyimide films (Kapton) (K/Au NHs-rGO), and the post coating of K/Au NHS with rGO can achieve higher temperatures with lower power density, while graphene has a marginal additional photothermal effect. In vivo experiments on subcutaneous infected mice showed that photothermal patch treatment promoted wound healing at the infected site.

**Figure 9:**
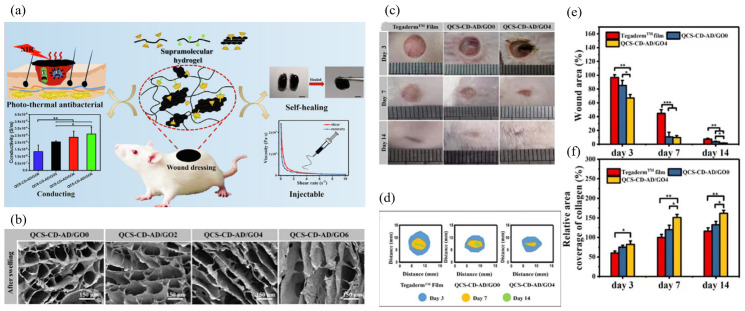
(**a**) Schematic diagram of hydrogel properties. (**b**) SEM of hydrogels after swelling. Scale bar: 150 μm. (**c**) Photographs of wounds on the 3rd, 7th, and 14th days for Tegaderm^TM^ film dressing, QCS-CD-AD/GO0 and QCS-CD-AD/GO4 hydrogels. (**d**) Schematic diagram of wound area during 14 days for the three groups. (**e**) Statistics of the wound area in each group (*n* = 5). (**f**) The statistical results of relative area coverage of collagen in the regenerated tissue, data for the group of Tegaderm^TM^ film on the 7th day were set as 100% (*n* = 5). * *p* < 0.05, ** *p* < 0.01. The QCS-CD-AD/GO hydrogels with GO-CD contents of 0.2 wt%, 0.4 wt%, and 0.6 wt% in the hydrogel were named QCS-CD-AD/GO2, QCS-CD-AD/GO4, and QCS-CD-AD/GO6, respectively. Data reproduced with permission from Ref. [79].

Hydrogels have a high moisturizing capacity, and hydrogel dressings can promote the proliferation and migration of fibroblasts by absorbing exudate from the wound area, accelerating epithelialization and wound healing [77]. To date, hydrogel dressings have been developed with various properties such as adhesion, antibacterial ability, antioxidant ability and drug release properties [78]. The unique physical and chemical properties of GBNs make them a good material to promote skin wound healing. Zhang et al. [79] formed antibacterial, injectable and conductive supramolecular hydrogels for wound repair by adding quaternary ammonium chitosan (QCS) and graphene oxide (GO) through the host-guest interaction between cyclodextrin (CD) and adamantane (AD) (Figure 9a). Compared with commercially available dressings (Tegaderm™ membranes, 3M company, South Dakota, USA) and unadulterated rGO hydrogels, hydrogels containing conductive component rGO significantly promoted in vivo full-layer wound healing, epidermal regeneration and granulation tissue thickening (Figure 9b–f). It increased the collagen area, decreased the interleukin-6 (IL-6) content, and up-regulated the expression of vascular endothelial growth factor (VEGF). GO can be added as an inorganic filler to the material, Yan et al. [80] prepared an irreversible heat-responsive hydrogel composed of poly(N-isopropyl acrylamide166-*co*-*n*-butyl acrylate9)-poly(N-isopropylacrylamide166-*co*-*n*-butyl acrylate9) (P(NIPAM166-*co*-nBA9)-PEG-P(NIPAM166-*co*-nBA9)PEP)and Ag@rGO nanosheet copolymer. Due to the addition of sufficient Ag@rGO nano-sheet layers, this composite hydrogel exhibits sol-gel irreversibility after formation at the skin wound, which can maintain a stable shape and adhere to the hand skin during outdoor exercise around 10 °C in winter, while PEP hydrogel transforms into a liquid form and is discharged from the skin. GO shows better water dispersion due to its hydrophilic groups, but it is much less conductive than graphene. However, under weakly alkaline pH conditions, dopamine can act as a reducing agent for GO to form rGO, increasing its conductivity while forming a hydrophilic polydopamine (PDA) coating on the surface of rGO by self-polymerization, enhancing its water dispersion [81]. Tang et al. [82] prepared rGO-PDA-CS/SF hydrogel scaffold by combining the dopamine-coated RGO-PDA (RGO-PDA) and the double crosslinked chitosan (CS)/silk fibroin (SF) network, which can transmit bioelectrical signals that promote cell growth. Meanwhile, the production of reduced and functionalized rGO-PDA not only improved the mechanical properties of the scaffold but also inhibited the excessive reactive oxygen species (ROS), which was beneficial to the regeneration of wound skin. Based on quaternary ammonium-chitosan (QCS), rGO-PDA and polyN-isopropyl acrylamide (PNIPAm), Li et al. [83] designed an injectable self-healing hydrogel as a multifunctional wound dressing, and the synthesis of rGO-PDA provides similar conductivity by near-infrared radiation, good photothermal properties and antibacterial properties enhanced, while IL-6 expression decreases, platelet endothelial cell adheres to the molecule-1(CD31) expression rises, enhancing cell adhesion and cell proliferation, accelerating the wound healing process. Based on rGO-PDA in the above literature, Tu et al. [84] added a modified antibacterial agent ε-polylysine(EPL) F127-EPL to the dopamine-modified GO hydrogel. GO’s high conductivity, angiogenesis promoting ability, and synergistic antimicrobial ability with EPL can quickly promote diabetic wound repair and skin regeneration. Liang et al. [81] successfully prepared a series of adhesion, hemostatic, antioxidant and conductive hydrogels containing hyaluronic acid-graft rGO-PDA. Due to the photothermal effect provided by rGO, the bacterial survival rate of wounds after 10 min NIR irradiation was very low (3.1%), which had a good effect on wound healing. Due to the different levels of in situ enzymes in wound exudates, Nguyen et al. [85] impregnated GO into a hydrogel of genipin cross-linked gelatin to control the release of GO enzymatically and found that the release rate of GO depends on the degree of crosslinking and environmental enzyme levels, and the enzymatic release of GO has uniform dispersion, which can promote the migration of human fibroblasts.

GBNs can also be synergistic with antibacterial materials. Norfloxacin (NFX) is a fluoroquinolone antibiotic with bactericidal activity, Ma et al. [86] prepared sodium alginate/GO/polyvinyl alcohol nanocomposite hydrogel. Because GO has a large number of -COOH, -OH and C=O groups, it can achieve adsorption of NFX through hydrogen bond and electrostatic interaction and maintain slow release of the drug. This avoids frequent dressing changes and ultimately promotes wound healing and prevents scar formation. Huang et al. [87] added Ag and GO to the hydrogel for synergistic antibacterial. The excellent near-infrared absorption and photothermal transformation ability of GO made hydrogel produce strong shrinkage under the irradiation of NIR, which was helpful to promote wound healing.

### 4.2. Muscle

Muscle tissue plays a crucial role in the human body; it can be divided into skeletal muscle, myocardial and smooth muscle. Skeletal muscles play a role in the movement and force of the body, and there are requirements for the elasticity of the material. The heart muscle has the effect of regulating the heart rate; it can transmit electrophysiological signals and has a more complex structure. Smooth muscle maintains vascular diameter and is sensitive to electrical stimulation [61,88,89]. However, many diseases and injuries, such as myocardial infarction (MI) and volume muscle loss (VML), can cause muscles, which are soft tissues, to be easily injured, not only to the pain of the patient but also to a lot of financial stress. Skeletal muscle is contracted tissue composed of highly directed and dense bundles of muscle fibers, which account for about 45% of the total weight [61], and physiologically can regenerate naturally after injury. However, once the loss of muscle mass reaches more than 20% of severe injury, endogenous self-repair becomes severely impaired, resulting in loss of muscle function [90]. Severe muscle trauma can lead to VML, fibrosis and scarring [91]. Unlike skeletal muscle, cardiomyocytes are terminally differentiated cells with very low self-regeneration ability [92]. Some cardiovascular diseases, such as MI, require heart transplants, but there is an extreme shortage of donors. Currently, surgical reconstruction is the most widely used treatment for severe muscle tissue injury. Unfortunately, this treatment approach has serious deficiencies in donor shortages, low survival rates, and complications in the supply area [93]. Therefore, muscle tissue engineering has become one of the most practical therapeutic strategies for muscle repair. Due to their excellent electrical conductivity and mechanical properties, GBNs have become an ideal candidate material for muscle tissue engineering scaffolds.

**Figure 10:**
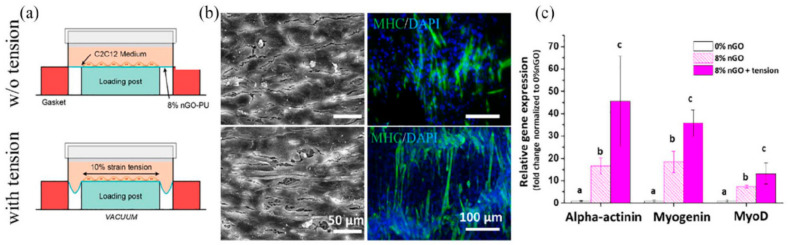
PU-nGO nanofiber synergizes with dynamic mechanical tension in myogenic differentiation. After 3 days of differentiation on 8% PU-nGO nanofiber under dynamic tension force using Flexcell machine (10% strain, 0.5 Hz, 1 h/day), (**a**) SEM, (**b**) immunocytochemistry of myosin heavy chain (MHC) images were taken. Fully covered C2C12 cells on 8% PU-nGO nanocomposite were observed by SEM. MHC expression and aligned myotubular formation were highly up-regulated in 8% PU-nGO under dynamic tension compared to static incubation. Dynamic tension and nGO worked in synergy to enhance the myogenesis of C2C12 cells. (**c**) Myogenic gene expression results (alpha-actinin, myogenin, and MyoD). Dynamic tension enhanced myogenic gene expression synergistically with nGO. Different letters indicating significant differences among them (*p* < 0.05, *n* = 4). Data reproduced with permission from Ref. [96].

GBNs show great potential in regulating skeletal muscle function due to their good biocompatibility, mechanical properties and electrical conductivity, which can promote cell attachment, proliferation and myogenic differentiation. Myoblasts play a crucial role in the growth of skeletal muscle. Shin et al. [94] modified C2C12 mouse myoblasts with nanofibers prepared by rGD-M13 phage and polylactic acid-glycolic acid copolymer (PLGA) and co-cultured with GO-containing medium. GO can significantly promote the growth and proliferation of C2C12 myoblasts and accelerate the process of myogenic differentiation. At the same time, the expression of myosin heavy chain (MHC) on the GO matrix composites was higher due to the better hydrophilicity and conductivity of the GO matrix composites. The effect of conductivity on muscle-form differentiation was also studied, and Jo et al. [95] obtained a reduced graphene oxide/polyacrylamide r(GO/PAAM) composite hydrogel by chemical reduction. The hydrogel has a young’s modulus of 50 kPa and conductivity of 10^−4^ S/cm, which is suitable for bonding with many soft tissues, including muscle. Compared with PAAM and non-reduced GO/PAAM, r(GO/PAAM) significantly promoted cell proliferation and myoblast differentiation. In addition, by applying electrical stimulation to conductive graphene hydrogels, the level of myogenic gene expression in myoblasts increased significantly compared with the unstimulated control group. For skeletal muscle engineering, composite materials can not only stimulate the myoblast differentiation of cells but also have the appropriate mechanical properties (such as flexibility), which can be used to adapt to different environments of muscle tissue. Jo et al. [96] developed a highly flexible composite nanofiber polyurethane-nGO (PU-nGO) scaffold through electrostatic spinning. The addition of GO improved the hydrophilicity, elasticity and stress relaxation ability of polyurethane, and PU-nGO nanofiber membrane enhanced the initial adhesion, spread and proliferation of C2C12 cells. Under dynamic force conditions, cells on PU-nGO nanofibers express significantly higher markers of myogenic differentiation at gene and protein levels and show more neatly arranged myotube formation, which is a potential matrix for future skeletal muscle engineering (Figure 10). Du et al. [97] developed poly citric acid-octanediol-polyethylene glycol-graphene nanocomposites (PCEG) with high elasticity, electrical conductivity and biodegradation. Without sacrificing hydrophilicity, the addition of rGO significantly improves the mechanical strength and electrical conductivity of the material, and the tensile strength and modulus of PCEG nanocomposites are increased by 200% to 300% and 400% to 800%, respectively, while maintaining good elasticity and elongation, in addition, PCEG can significantly promote the attachment, proliferation, myogenesis differentiation and in vivo skeletal muscle tissue repair.

The myocardium has the function of transmitting electrophysiological signals, and GBNs can be used as tissue engineering materials due to their good electrical conductivity. Jiang et al. [98] designed a chitosan-GO conductive scaffold with a conductive and porous structure, which has high conductivity (0.134 S/m) close to that of natural heart tissue and improved cell survival rate, cell adhesion and intercellular network formation ability of cultured heart H9C2 cells in vitro. In addition, heart-specific genes and proteins expressions associated with muscle contraction and electrically coupled electrical conduction, such as connexin-43 or cardiac troponin (cTnT), were upregulated. In addition to scaffolds, hydrogel structures are also good tissue engineering materials. Shin et al. [99] introduced rGO into gelatin methacryloyl (GelMA) hybrid hydrogels, exhibiting enhanced electrical conductivity and mechanical properties. Cardiac precursor cells cultured on this hydrogel showed improved survival and proliferation, with stronger contractibility and faster spontaneous pulse rate. Results showed that rGO-GelMA culture had a well-defined, more uniaxial sarcomere structure and uniformly distributed Cx-43 compared to original GelMA. Cardiomyocytes play an important role in the tissue engineering of the myocardium [100].

### 4.3. Nervous

The nervous system maintains body balance by controlling and regulating the different activities of organs [101]. It is divided into the central nervous system (CNS) and the peripheral nervous system (PNS) [102]. The CNS is made up of the brain and spinal cord, while the PNS is made up of motor neurons and sensory neurons that can transmit signals between the CNS and the rest of the body [103].CNS damage includes traumatic injuries (such as falls, car accidents and attacks) or non-traumatic injuries (such as neurodegenerative diseases, strokes and brain tumors) [104]. Neurodegenerative diseases such as Alzheimer’s disease, Parkinson’s disease, etc. Although there are some effective drugs to control or slow down the process of these diseases, most of them are irreversible or incurable, and their incidence is increasing with the aging of the population [105]. In addition, due to the widespread presence of nerves throughout the body, peripheral nerve injury (PNI) has been reported as an important cause of clinical disability [106]. When the injury occurs in patients with this disease, the signal transmission process may be partially or completely interrupted. This deficit in sensory and motor function faces not only sensory loss and motor disorders but also triggers neuropathic pain and cold intolerance, which has a great impact on their quality of life [107]. Although neurons are the most important part of these two nervous systems, they cannot undergo mitosis, supporting the limited ability of glial cells to divide, resulting in slow/inability to heal after nervous system injury [108]. Autologous nerve transplantation is a commonly used treatment for nerve injury however, limited nerve graft availability, the incidence of donor sites, increased risk of infection due to surgery, and immune response are limiting issues of treatment. Neural tissue engineering is considered the best alternative, so scaffolds for neural tissue engineering should mimic the natural extracellular matrix (ECM) to provide not only mechanical support for tissue regeneration, but also modulate biological signals to guide axon growth, promote regeneration, and stimulate integration into existing healthy tissues [14,104]. Since the nervous system communicates neurons with other types of cells through electrical signals, the excellent conductivity and biocompatibility of GBNs can provide an excellent platform for stimulating neurons. At the same time, GBNs have a unique surface structure and negligible cytotoxicity, making them ideal for neural tissue engineering. In addition, GBNs are often coupled to neurotransmitters, anticoagulants, and growth factors for repairing or regenerating neural structures.

Studies have shown that GBNs can greatly promote the migration, proliferation and differentiation of nerve cells and can be used for nerve regeneration. Feng et al. [109] studied the effect of GO and rGO membrane on the differentiation of adipose stem cells (ADSCs) into nerve cells, and the effect of GO inducing ADSCs to differentiate into neurons was more significant after seven days of culture. Ma et al. [110] prepared 3D hybrid graphene (3D-HG) containing a 3D skeleton and 2D thin films by chemical vapor deposition (CVD) using Cu/Ni hybrid template as catalysts, and the excellent structural controllability of 3D-HG was conducive to the gap between the differentiated neural precursor cells (NPCs) connecting the backbone and promoting the formation of neural networks. It was also found that the size effect of the graphene scaffold affected nerve cell behavior, that is, the narrower the width of the skeleton, the smaller the elastic modulus, because this softer substrate increased the promotion effect of neural stem cells to neuronal differentiation. Wychowaniec et al. [111] developed rGO-MXene hydrogel by reacting with GO by MXene (Ti3C2Tx) and using ethylenediamine for inter-slice crosslinking induction. rGO-MXene hydrogels are more hydrophilic, softer, and have a unique porous structure than rGO hydrogels. Their results suggest that rGO-MXene hydrogels can enhance neuron-like cell adhesion (SH-SY5Y cells), promoting the formation of three-dimensional cell networks. Niu et al. [112] prepared a thin film consisting of silk fibroin (SF) and graphene, in which rat-induced pluripotent stem cells (IPSCs) were cultured. They found that because graphene can transmit electrical stimulation signals and stimulate neuronal growth, the graphene/SF membrane can promote the neural differentiation process of IPSCs, and the degree of neural differentiation increases with the increase of graphene content. The reduction will enhance the conductivity of the material. Magaz et al. [113] add a 10 wt% concentration of GO to SF and perform in situ reductions to obtain the electroactive SF/rGO nanofiber scaffold. With the increase of filler content, surface roughness and protein adsorption capacity tend to increase. Compared with SF/GO samples, the reduction treatment greatly enhances the conductivity of the scaffold and improves the proliferation rate of neuronal NG108-15 cells, promoting protrusion growth.

**Figure 11:**
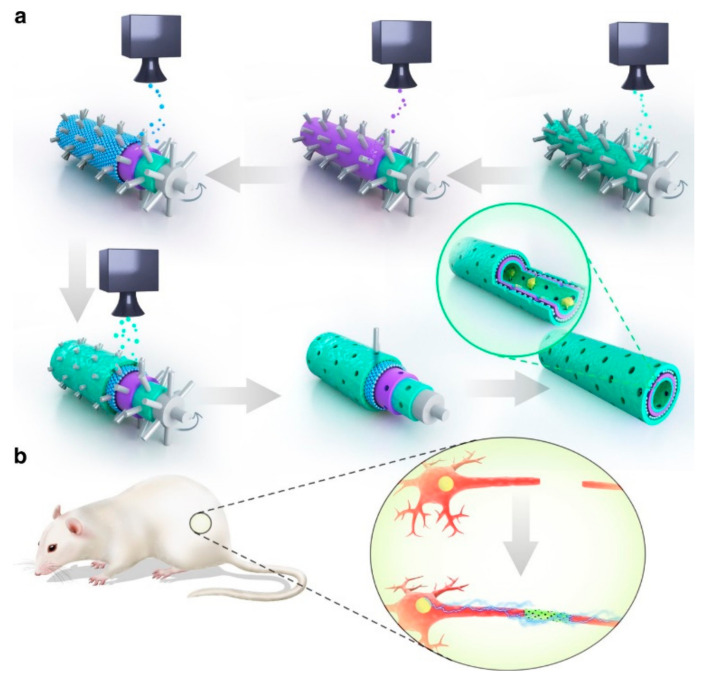
Schematic diagram of graphene neural catheter made by LBLC method: (**a**) The innermost and outermost green layers are the PDA/RGD hybrid layers. The purple layer is a mixed layer of single or multilayer graphene and PCL. The blue layer is a repeat of the graphene and PCL mixed layer. (**b**) Single or multilayer graphene/PCL nerve catheterization in an SD rat model of sciatic nerve defect. Data reproduced with permission from Ref. [116].

Treatment based on neural stem cells (NSCs) is a promising candidate for the treatment of neurodegenerative diseases, Huang et al. [114] embedding NSCs in hydrogels prepared after mixing graphene or GO with polyurethane (PU) by 3D bioprinting techniques. This hydrogel containing very low content (25 ppm) graphene nanomaterials has suitable rheological properties, improving cell survival and oxygen metabolism (increasing by 2–4 times). Moreover, the NSCs embedded in graphene/PU or GO/PU have obvious β-tubulin and glial fibrous acidic protein (GFAP) expression, while the expression of NSCs is not seen in PU, which effectively promotes neural stem cell differentiation. Schwann cells (SCs) are an important part of nerve regeneration, promoting axon growth by secreting various growth factors. Wang et al. [115] coated GO on the electrospun composite Antheraea pernyi silk fibroin (ApF)/(Poly(L-lactic acid-*co*-caprolactone)) (PLCL) scaffold to obtain GO-ApF/PLCL scaffold, the addition of GO not only improved the mechanical properties of ApF/PLCL scaffold but also improved the hydrophilicity of stent. The GO-coated ApF/PLCL scaffold can significantly promote SC migration, proliferation and myelination, induce PC12 cell differentiation, and up-regulate the expression of focal adhesion kinase (FAK) in PC12 cells. In vivo, this scaffold successfully repaired a 10 mm sciatic nerve defect and showed a similar healing ability to autologous transplantation. Qian et al. [116] use 3D printing and layer-by-layer casting (LBLC) techniques to encapsulate polydopamine/arginylglycylaspartic acid (PDA/RGD) in a scaffold consisting of graphene and polycaprolactone (PCL) (Figure 11). The innermost and outermost layers of RGD and PDA favor cell adhesion and proliferation. Graphene/PCL bilayers can provide a certain stiffness conducive to long-term use. This scaffold can both conduct bioelectrical signals and promote nutrient exchange and waste excretion through porous structures. Loading Sherwang cells to further enhance their effects, compared with other scaffolds without any one component. The sciatic nerve recovery of Schwann cell-loaded nanoscaffold is faster, suggesting that the synergy of graphene, PCL, adhesion molecules and cells is a promising peripheral nerve repair method. Acetylcholine is a neurotransmitter that has neuromodulatory functions, and phosphorylcholine is a platelet activator that is involved in the regulation of a variety of cellular functions. Tu et al. [117] constructed an intelligent bionic GO matrix composite by covalently bonding acetylcholine groups (dimethylaminoethyl methacrylate, DMAEMA) or phosphoruscholine-like groups (2-methacryloyloxyethyl phosphorylcholine, MPC) to promote the germination and growth of neural protrusions. On the 2nd and 7th days after cell inoculation, the number and average length of neurites in the GO-DMAEMA and GO-MPC composites were significantly higher than those in control GO. Netrin-1 is a classic axon-guiding molecule that also has a positive effect on angiogenesis. Huang et al. [118] synthesized a 3D graphene mesh tube (GMT) and subsequently filled it with alginate, and a gelatin-methacryloyl (GelMA) prepared with a double network (DN) hydrogel. The hydrogel has a suitable mechanical strength (Young’s modulus 725.8 ± 46.52 kPa) and good conductivity (6.8 ± 0.85 S/m), GMT/DN hydrogel can support the proliferation and arrangement of RSC96 nerve cells compared with pure DN hydrogels due to the good electrical activity and molecular interaction of GMT. Importantly, when the concentration of netrin-1 is 100 mg/mL, it can promote the migration of Schwann cells and the tubular shape of human umbilical vein endothelial cells (HUVECs). The GMT/DN hydrogel scaffold loaded with netrin-1 can effectively promote peripheral nerve regeneration.

Although there are many excellent properties in the developed composites (such as biocompatibility, biodegradability, nerve conduction, and suitable surface and mechanical properties), there are still some complications such as inflammatory response, oxidative stress, fibrosis or scar tissue, and appropriate vascular formation and deficiency that affect the success of regeneration. Therefore, these problems must be overcome to achieve successful neural repair. Qian et al. [119] prepared GO/PCL nano-scaffolds using an integrated molding method. The stent promotes angiogenesis through the AKT-endothelial nitric oxide synthase (ENOS)-vascular endothelial growth factor (VEGF) signaling pathway, which in turn promotes PN regeneration. In addition, brain damage caused by nerve implants can trigger a neuroinflammatory response that can cause hypoxia, ischemia, viral, and bacterial infections [120], and astrocytes, peripheral macrophages, and microglia are the primary counterparts for this response [121]. Song et al. [122] observed that 3D graphene supports the growth of microglia and exhibits good biocompatibility. Durso et al. [123] produced a new interface to improve the interaction between GO and astrocytes. By atom transfer radical polymerization (ATRP), phospholipid (PL)was partially grafted onto GO by polymer brush between acryloyl modified PL and brominated initiator modified GO nanosheets. Primary cultured rat cortical astrocytes have approximately 3-fold increased adhesion on the GO-PL matrix compared to glass matrix and non-functionalized GO matrix coated with standard adhesions (i.e., poly-D-lysine, PDL). Additionally, astrocytes on GO-PL did not show significant glial reactivity, indicating that the material interface did not cause a harmful inflammatory response when interacting with astrocytes.

### 4.4. Blood Vessel

Blood is transported from the heart through a hierarchical arrangement of blood vessels, from the arteries to the arterioles, and finally to the capillary network, forming a compact closed cycle that penetrates most of the body’s tissues and the exchange of oxygen, nutrients, and waste products [124]. The arteries, veins, and capillaries have an inner membrane consisting of endothelial cells (EC), which is responsible for antithrombotic aspects; the arteries and veins are further bound to the second layer of the median membrane, which is responsible for mechanical strength; and the outer membrane provides vascularization and autonomic control [125,126]. Damage to the function of blood vessels (BV) can occur in these three types of blood vessels. In the large vascular system (BV inner diameter >1 mm), ischemic diseases such as atherosclerotic cardiovascular disease (CVD) are prone to occur, resulting in BV occlusion. In the middle vessel (50 μm < BV inner diameter < 1 mm) and microvascular (BV inner diameter < 50 μm), occlusion can lead to tissue ischemia, and in severe cases, complete tissue dysfunction requiring organ replacement [124]. Traditional vascular repair is performed with autologous transplantation, but the options available for grafted vascular replacements are very limited, and additional associated surgery may result in high incidence and failure rates at donor sites [127]. To overcome the limitations of these current treatments, vascular tissue engineering has become an effective way to produce a variety of potentially functional vascular alternatives. It reconstructs the structure and function of extravasal blood vessels before transplantation by designing multi-materials to mimic the extracellular matrix of blood vessels [128]. Current vascular tissue engineering should meet the following key requirements: (1) the size of tubular tubing (lumen diameter, length, and length) must match the application; (2) the mechanical properties of the graft and vessel must match, at least within the limit tensile strength range; (3) the graft’s thrombotic capacity must be low (enhanced hemocompatibility); and (4) regenerative/integration potential to ensure the graft’s longevity [126]. GBNs, with their unique physicochemical properties, have aroused people’s research interest in applying them to vascular tissue engineering.

Alavije et al. [129] prepared polyvinyl alcohol (PVA) flat and tubular nanocomposite scaffolds containing 1, 2 and 3 wt% graphene by electrospinning method, and when the amount of graphene was added to 3 wt%, the elongation at break of the scaffold was improved, the water contact angle increased to 69°, and the adhesion and proliferation tendency of endothelial cells on the surface of the scaffold were enhanced. At the same time, compared with flat stents, tubular stents improve electrical performance. GO not only promotes cell growth but also improves the thermomechanical properties of the polymer matrix. Shin et al. [130] prepared RGD peptide GO co-functionalized poly (lactide-co-glycolide, PLGA) nanofiber membrane (RGD-GO-PLGA), which can provide an ideal microenvironment for the growth of vascular smooth muscle cells. Due to the synergistic effect of RGD peptides and GO, the initial attachment and proliferation of vascular smooth muscle cells on the RGD-GO-PLGA nanofiber membrane has increased significantly, which is expected to become a tissue-engineered scaffold material that effectively promotes the regeneration of vascular smooth muscle. rGO’s unique two-dimensional structure and high specific surface area provide a suitable geometric configuration as a catalyst carrier, and Huo et al. [131] successfully constructed rGO enzyme-coated small-bore tissue-engineered vessels (TEBV) with antiplatelet and endothelial functions. After seven days of transplantation, the patency rate of TEBV reached 90%, ensuring its antithrombotic function and patency.

## 5. Conclusions

Due to their diverse properties, GBNs have made remarkable progress in synthesis and functionalization, which opens up a new way to explore their application in tissue engineering. In this paper, the structure and properties of GBNs, the improvement of biocompatibility, and their physical and chemical properties in soft tissue engineering are reviewed and discussed in detail. GBNs’ excellent mechanical strength, hardness, conductivity, and various 2D and 3D morphologies can stimulate stem cell proliferation and differentiation into specific lineages, including skin, nerves, muscles, heart, and blood vessels, making them potential candidates for tissue engineering. GBNs have good photothermal properties, and antibacterial activity can be applied to the wound site to prevent infection and delay the wound healing process. The large specific surface area of GBNs, that react with rich active groups for functionalization, can improve biocompatibility, reduce toxicity, and have special properties such as targeting. In addition, pH-sensitive drug release therapy can be achieved depending on its inherent pH responsiveness and the pH difference between normal and tumor cells.

Despite some achievements, there are still some challenges and difficulties in the study of GBNs in biological systems. Firstly, the aggregation of graphene in solution and the uneven distribution of graphene nanosheets in the matrix affects the practical application of materials, and new methods must be developed to prevent the aggregation of graphene. Secondly, most of the research results put forward a controversial view of the antibacterial mechanism of GBNs, and a deep understanding of the relevant mechanisms and influencing factors of their antibacterial activity is a question worthy of further study. Thirdly, while the toxicity of GBNs can be greatly reduced through chemical functionalization, potential long-term toxicity still has a large impact on human health and ecological risks. Therefore, more toxicity studies using in vivo animal models are needed to investigate the safety and biocompatibility of GBNs materials. Fourth, considering the problem of large-scale production and clinical application, a large number of experimental studies are still needed. In summary, despite the various outstanding issues and challenges, the use of GBNs may provide a breakthrough opportunity for future tissue engineering applications.

## Figures and Tables

**Table 1:** Summary of GBNs for soft tissue engineering.

Soft Tissue	Function	Advantage	Suitable for	Referents
Skin	Feel the external environment and dissipate heat	Promotes wound healing while providing the bactericidal ability	Traumatic injury, burns, surgery, non-healing ulcers and chemical injury	[60]
Muscle	The skeletal muscle is responsible for movement and force in the body; the heart muscle regulates the heart; the smooth muscle maintains vascular diameter and is sensitive to electrical stimulation rate	Good biocompatibility, mechanical properties and electrical conductivity	Myocardial infarction and mass muscle loss	[61]
Nervous	To maintain balance by controlling and regulating the different activities of the organs	Promote the migration, proliferation and differentiation of nerve cells	Central nerve injury includes falls, car accidents, neurodegenerative diseases, stroke, etc.; peripheral nerve injury can cause sensory loss and motor impairment	[62]
Blood vessel	Transport blood and exchange oxygen, nutrients and waste in tissues	Mimicking the extracellular matrix to provide mechanical properties suitable for vascular stretching	Atherosclerotic cardiovascular disease and other ischemic diseases, resulting in vascular occlusion	[63]

## Data Availability

No new data were created or analyzed in this study. Data sharing is not applicable to this article.
